# The Influence of Follicular Fluid Metals on Assisted Reproduction Outcome

**DOI:** 10.1007/s12011-023-03578-3

**Published:** 2023-02-18

**Authors:** Rubí Rodríguez-Díaz, Raquel Blanes-Zamora, Soraya Paz-Montelongo, Jorge Gómez-Rodríguez, Sonsoles Rodríguez Fiestas, Dailos González-Weller, Ángel J. Gutiérrez, Carmen Rubio, Arturo Hardisson, Daniel Niebla-Canelo, Samuel Alejandro Vega, Enrique González-Dávila

**Affiliations:** 1 Human Reproduction Unit, Hospital Universitario de Canarias, La Laguna, Tenerife Spain; 2 Obstetrics and Gynecology, Universidad de La Laguna, Tenerife, Spain; 3 Toxicology, Universidad de La Laguna, Tenerife, Spain; 4 Mathematics, Statistics and Operations Research. IMAULL, Universidad de La Laguna, Tenerife, Spain

**Keywords:** Metal, Follicular fluid, ART, Embryo quality, Pregnancy, Polycystic ovary syndrome

## Abstract

Infertility has become more common, with an increased exposure to toxic compounds including heavy metals (HM). Follicular fluid (FF) surrounds the developing oocyte in the ovary and can be analysed to assess metal content. The levels of twenty-two metals were measured in the FF of ninety-three females in a reproduction unit, and their influence on assisted reproduction technique (ART), were examined. The metals were determined by optical emission spectrophotometry. Low values of copper, zinc, aluminium, and calcium favour polycystic ovary syndrome. The relationships between the number of oocytes and metals: iron (*r*_*s*_=0.303; *p*=0.003) and calcium (*r*_s_=−0.276; *p*=0.007) are significant, as well as between the number of mature oocytes with iron (*r*_s_=0.319; *p*=0.002), calcium (*r*_s_=−0.307; *p*=0.003) and sodium (*r*_s_=−0.215; *p*=0.039) and are near to significance in the case of aluminium (*r*_s_=−0.198; *p*=0.057). In the group with a fertilisation rate ≤ 75%, 36% of the women presented calcium >176.62 mg/kg compared to the group with a fertilisation rate ≥ 75% where this percentage was only 10% (*p*=0.011). An excess of iron and calcium reduces the good quality embryo rate, and an excess of potassium impairs the blastocyst rate. If potassium is above 237.18 mg/kg and calcium is below 147.32 mg/kg, these conditions favour embryo implantation. Pregnancy is influenced by high potassium and low copper levels. Controlling exposure to toxic elements is recommended for all couples with reduced fertility or receiving an ART.

**Supplementary Information:**

The online version contains supplementary material available at 10.1007/s12011-023-03578-3.

## Introduction

Fertility is currently decreasing, and reproductive disorders are affecting a growing number of couples [1]. One of the factors that may be responsible is toxic metal environmental contamination leading to the detection of the negative effects of some toxins such as heavy metals (HM) [2]. Their accumulation in reproductive tissues corroborates the possibility of their procreative toxicity [3], and the worst consequences are observed in females, since the number of germ cells present in the ovary is fixed during foetal life which are not renewable [4], and this decreases over time.

The following are the main mechanisms of HM toxicity: generation of free radicals causing oxidative stress (OS) in body cells through lipid membrane disintegration [5], and damage of biological molecules such as enzymes, proteins, lipids, and nucleic acids, and to DNA [6]. Toxic metal exposure increases the content of reactive oxygen species (ROS) in the human body and eventually induces a series of oxidative damage processes [7], this disturbs the antioxidant defence system, thus promoting OS, necrosis, and apoptosis [8]. HM can bind to oestrogen receptors and may interfere with hormone signalling pathways, causing sexual hormone disruption [9].

Exposure to metals is associated with oocyte maturation [5], ovulation, and fertilisation [10], and therefore assisted reproduction treatments (ART) are affected [2]. If the oocyte develops in a toxic environment that disturbs the primordial follicles, the maturation and quality of the embryo is harmed, and this could be one of the effects of HMs [5, 11].

Bloom et al. [12], found an association between the decrease in fertility, with high doses and professional exposures to Hg, Cd, and Pb, and Ingle et al. [13], observed a negative association between the presence of Cr in follicular fluid (FF) and the number of mature oocytes recovered, and Zn with the fertilisation rate (FR). When these determinations were made in urine, it was found that the higher the levels of Co, Cr, Cu, Mn, and Mo were associated with more oocytes recovered per woman.

Other authors reported that a higher concentration of HM was associated with low-quality oocytes and embryos [14], and on the contrary, it was observed that a higher level of Zn in serum and FF could have a positive role in ART [15]. Although their mechanisms and concentrations are unknown, Pb in blood and Cu in FF seem to have significant impacts on the outcome of the ART cycle, but further studies with larger cohorts and in different patient populations are needed to define their effects on oocyte quality, embryo development, and human reproduction in general [3].

Thus, oocyte quality is not only influenced by the nuclear and mitochondrial genome, but also by the microenvironment provided by the ovary and the preovulatory follicle that influences transcription and translation, and consequently, cytoplasmic maturity [16]. The oocyte is immersed in FF inside the ovarian follicle and is the oocyte’s first exposure to environmental contaminants [17]. Human FF is composed of ultra-filtered blood plasma exudates and granulosa and theca ovarian cell secretions. Since ovarian vessels do not penetrate the follicle basal lamina, FF directly provides the microenvironment of granulosa cells and oocytes. Nutrients and exogenous compounds in blood can enter the follicle antrum by passing through the basal lamina [18].

Therefore, the present study aims to examine the association between the presence of twenty-two metals in FF and the ART outcomes in terms of fertilisation rate, embryo quality, implantation, and pregnancy rates.

## Material and Methods

### Samples

A prospective study was conducted with ninety-three women seeking ART in the Reproduction Unit of the Hospital Universitario de Canarias, between February 2019 and December 2020, who underwent a metal detection procedure in FF.

All subjects agreed to participate in the study and signed a written informed consent form for all procedures to be carried out. The study was approved by the Ethics Committee of the Hospital Universitario de Canarias (Reg. No. CHUC 2018 53). Participant recruitment and clinical procedure has been previously described in detail [19].

The employment categories of the participants are as follows: health 15% (nursing assistants, caregivers, nurses, beauticians, pharmacists, masseuses, doctors, and social workers), administrative 31% (lawyers, office workers, quantity surveyors, bank employees, accountants, graphic designers, business women, civil servants, real estate agents, teachers, and secretaries), farming 3% (foreman and packaging staff), transport and retail 28% (airport staff, flight attendants, shop, and supermarket assistants), hospitality 19% (waitresses, chambermaids, cooks, cleaning staff, receptionist, and restaurant staff), and others 4% (unemployed and students).

### Assisted Reproduction Treatment

The participant underwent a puncture and aspiration of the ovarian follicles for oocyte extraction with assessment of oocyte presence and for the analysis of metals in the FF.

A controlled ovarian stimulation (COS) to retrieve the oocytes was performed. The following antagonist protocol was used: pharmacological stimulation with recombinant gonadotropins began on day two of the cycle, with the administration of a variable dose of 225–300 IU of rFSH (Puregon®, Organon, France or Gonal-F®, Merck Serono, France) associated or not with 100–150 IU of urinary gonadotropin HMG (Menopur®). Once the main follicle reached 14 mm. in diameter, the GnRh antagonist was added subcutaneously, daily, starting with 0.25 mg of ganirelix or cetrorelix (Ganirelix, Orgalutran®, Organon, France; Cetrorelix, Cetrotide®, Serono, France). When the follicles were at least 17 mm, final maturation was carried out with rHCG (Ovitrelle® 250 microgram solution for injection in a pre-filled pen, choriogonadotropin alfa (Merck Serono, Bari, Italy).

The ovum pick up (OPU) was performed thirty-six hours after the administration of rHCG, and the oocyte was obtained via transvaginal needle under ultrasound vision. The FF is separated from the oocyte during processing and is routinely collected and discarded during ART but can be retained for analysis.

The FF of several follicles of each patient, both small and large, was pooled and stored in the fridge until the metal determination. Once the oocytes had been retrieved, a semen sample was collected on the day of the OPU and subsequently processed for the ART procedure. The oocytes were then inseminated, either by IVF or ICSI technique according to indication, prior to fertilisation and subsequent embryo division taking place.

Fertilisation rate (FR) was assessed at eighteen hours post insemination and was defined as the percentage of fertilised oocytes with respect to the number of mature oocytes, provided at least one had matured (MII).

Embryo cleavage rate (CR) was established as the number of divided embryos with respect to fertilised embryos, provided that at least one had been fertilised. A good prognosis for CR was 100% of the embryo cohort. Good embryo quality (GEQ) was established according to the ASEBIR (Association for the Study of the Biology of Reproduction) parameters for ART cycles. Embryo quality grades A and B on day three of division were considered good quality embryos [20]. A cycle is considered of good prognosis when the value of GEQ is greater than 66.6% of the total embryo cohort.

The achieved blastocyst rate (BR) was also considered a measure of the degree of the cycle’s quality. Out of the total number of cycles, sixty-one had at least one cultured embryo until days 5–6, and the cycle was considered of good prognosis when at least 50% of the cultured embryos reached the blastocyst stage.

The embryo implantation rate (IR) was defined as the percentage of embryos transferred to the uterus resulting in a pregnancy. Pregnancy rate (PR) was defined as the number of cycles with at least one gestational sac among the number of cycles with embryo transfer (ET); 106 fresh ET were performed in a total of fifty-five patients and sixty-six frozen embryo transfers (FET) in a total of thirty-six patients.

The study group was divided into two groups based on the ART outcome. Special attention was paid to patients diagnosed with PCOS. The primary aim of the study was pregnancy and live birth achieved, and the secondary aims were to obtain the FR, CR, PR, and GEQ.

### Treatment of the Samples

**Fig. 1:**
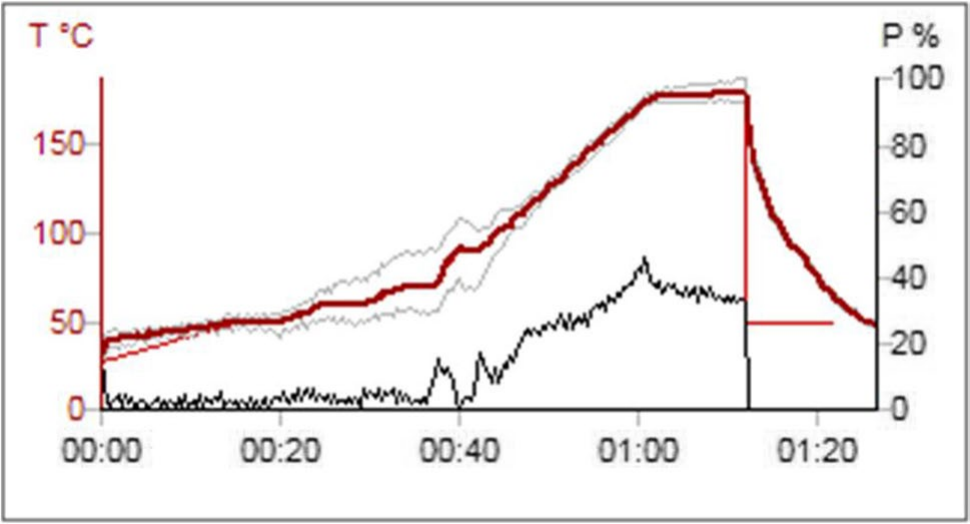
Digestion process. Graphic temperature (°C) vs time (hh:mm)

Sample processing was performed at the Toxicology Department of the University of La Laguna and in the Legal and Forensic Medicine Institute. Ninety-three samples were processed in triplicate by microwave digestion (Multiwave Go, Antonpaar). A total of 1 mL of sample, 2 mL of H_2_O_2_ 30% (Honeywell, Fluka), and 4 mL of HNO_3_ 65% (Honeywell, Fluka) were added to each reactor which were then submitted to the digestion process. This process lasted 1 and 26 min and reached a maximum temperature of 180°C (Fig. 1). After digestion, the reactor contents were made up to 10 ml with distilled water (Milli-Q Gradient A10, Millipore, MA, USA) for analysis.

**Table 1:** Limits of detection (LOD) and quantification (LOQ) for each level of follicular fluid metal

Metal	Wavelength (nm)	LOD mg/L	LOQ mg/L
Al	167.0	0.005	0.015
B	249.6	0.008	0.027
Ba	455.4	0.0006	0.002
Ca	315.8	1.629	5.432
Cd	214.4	0.0007	0.002
Co	228.6	0.001	0.005
Cr	267.7	0.001	0.005
Cu	324.7	0.003	0.011
K	766.4	1.764	5.883
Li	670.7	0.013	0.031
Mg	383.8	1.580	5.268
Mn	257.6	0.0008	0.003
Mo	202.0	0.0016	0.005
Na	818.3	2.221	7.404
Ni	221.6	0.0009	0.003
Pb	220.3	0.0009	0.003
Sr	407.7	0.003	0.011
V	292.4	0.0014	0.004
Zn	213.8	0.0027	0.009

Metal detection was conducted in the Canary Health Service which is in association with the Toxicology Department of the University of La Laguna and in the Legal and Forensic Medicine Institute. The following metals were measured in the FF: aluminium (Al), boron (B), barium (Ba), calcium (Ca), cadmium (Cd), cobalt (Co), chromium (Cr), copper (Cu), iron (Fe), potassium (K), lithium (Li), magnesium (Mg), manganese (Mn), molybdenum (Mo), sodium (Na), nickel (Ni), lead (Pb), silica (Si), tin (Sn), strontium (Sr), vanadium (V), and zinc (Zn).

The samples were analysed using an inductively coupled plasma–optical emission spectrometer (ICP-OES), model ICP-OES Thermo Scientific iCAP PRO (Waltham, MA, USA) with an automatic Auto Sampler. The instrumental limits of detection and quantification were estimated based on the instrumental response of the instrument. Specifically, they were determined by analysing fifteen blanks under reproducible conditions [21]. The limits of detection and quantification for each level of FF metal are shown in Table 1.

### Data Analysis

Data were summarised as relative frequencies for categorical variables, means ± standard deviation for normally distributed variables, and medians (interquartile range IQR, P25; P75) for non-normal data. Comparisons were performed using Pearson’s chi-square test, Kruskal-Wallis test, or Mann Whitney *U* test and ANOVA or *t*-student according to the type of variable and number of groups to be compared. The degree of relationship between continuous variables was calculated using the Pearson or Spearman correlation coefficient according to their distributions. The cut-off points or threshold of metal content to discriminate between the success of the different reproductive phases was established by applying receiver operating characteristics (ROC) curves and choosing the point according to the Youden index criterion. Subsequently, logistic regression with Wald’s backward variable selection method was used to model success in the different phases including variables from the spermiogram, as well as metal content. ROC curves and the area under the curve (AUROC) were included. A generalised linear mixed model with binary logistic distribution and logit link, including random intercept and slope, was applied in the study of embryo implantation to consider couples with different numbers of interventions. The selection of variables was made using a backward type of procedure (p-out = 0.10) to prevent a possible multicollinearity effect. The value of Akaike information criterion (AICc) and percentage of concordance was provided. SPSS V 25 (IBM SPSS Statistics) and MedCalc V 19.5 (MedCalc Software Ltd.) were used. A value of *p* ≤ 0.05 was considered significant.

## Results

### Demographic and Clinical Characteristic

The participants were between twenty and forty-two years of age and they were all living in the Canary Islands, Spain. The mean age of the patients was 36.0 ± 3.9 years, and the body mass index (BMI) was 24.7 ± 3.3 kg/m^2^.

Among the participants, 80.4% had primary infertility, 3.2% secondary, and 8.7% reported previous pregnancy loss. Another 5.4% attended as a woman without a partner, and 2.2% as a woman with a female partner. Regarding the pathology of the participants, 24% had PCO, 8.7% had a tubal pathology, 5.4% had fibroids, 3.2% had endometriosis, and cervical pathologies, in the same proportion. All the patients followed the Mediterranean diet and only three participants had mild exposure to toxicants as farmers, such as packaging staff. We recorded toxic habits, such as tobacco (17.4% of the participants) or occasional intake of alcohol (27%). Regarding the place of residence, 65.6% lived in the island of Tenerife, 10.7% in La Palma, 10.7% in Lanzarote, 10.7% in Fuerteventura, 1.07% in La Gomera, and 1.07% in El Hierro.

The male participants had a mean age of 38.2±5.2 years, and a mean BMI of 27.4±4.3 kg/m^2^.

### FF and Metals

The concentration of metals in the FF samples varied depending on the metal. The metals that had concentrations above the instrumental LOQ to the greatest extent were Na (97.83%), Fe (95.65%), and K (93.47%), while Co, Mo, Cr, B, Cd, Li and V, Si, Hg were not detected in any FF sample of the cohort. And those samples where the signals were lower than the instrumental LOQ were not considered in the statistical analysis. This does not mean they were not present, as they could have been below the detection limit of the device (ICP-OES), which does not rule them out as being potentially harmful [17].

Mean concentration (mg/kg) was highest for the macroelement metals Na, K, and Ca, followed by essential metals Fe, Zn, Cu, and Mn. The non-essential metals Ba and Al were detected (Table 2).

**Table 2:** Metal content in follicular fluid

Metals *n* (%)	*N* > LOQ	% >LOQ	mg/kg
Essential metals			
Cu	85	92.39	0.88 ±.0.30
Fe	88	95.65	15.48 ± 13.89
Fe*	–	–	11.2 (7.0;17.8)
Zn	85	92.39	1.14 ± 0.39
Mn	58	63.04	0.04 ± 0.01
Non-essential metals			
Ba	11	11.96	0.06 ± 0.07
Al	65	70.65	0.66 ± 0.66
Al*	–	–	0.50 (0.32;0.80)
Ni	11	11.96	0.31 ± 0.41
Pb	2	2.17	–
Sr	1	1.09	–
Macroelements			
Ca	85	92.39	148.51 ± 49.24
K	86	93.48	332.47± 118.73
Mg	10	10.87	68.80 ± 6.75
Na	90	97.83	4550.45± 1299.24

*Median (interquartile range)

### Metal Correlation

K was positively correlated with Fe and Zn, and was significant with Na (*r* =0.7389; *p* < 0.001); Cu was positively related to Zn, Ba, Ca, and Na (the highest correlation was with Ba); and Mn was positively correlated with Zn. Ba was positively correlated with Cu and Zn.

In addition, Al was positively correlated with Zn and Mn. Na was positively correlated with Cu, Ca, and K, and Zn correlated positively with Mn, Ba, Al, and K (it should be noted that the ratio of Zn and Al may be conditioned by the excessive value of Al).

**Fig. 2:**
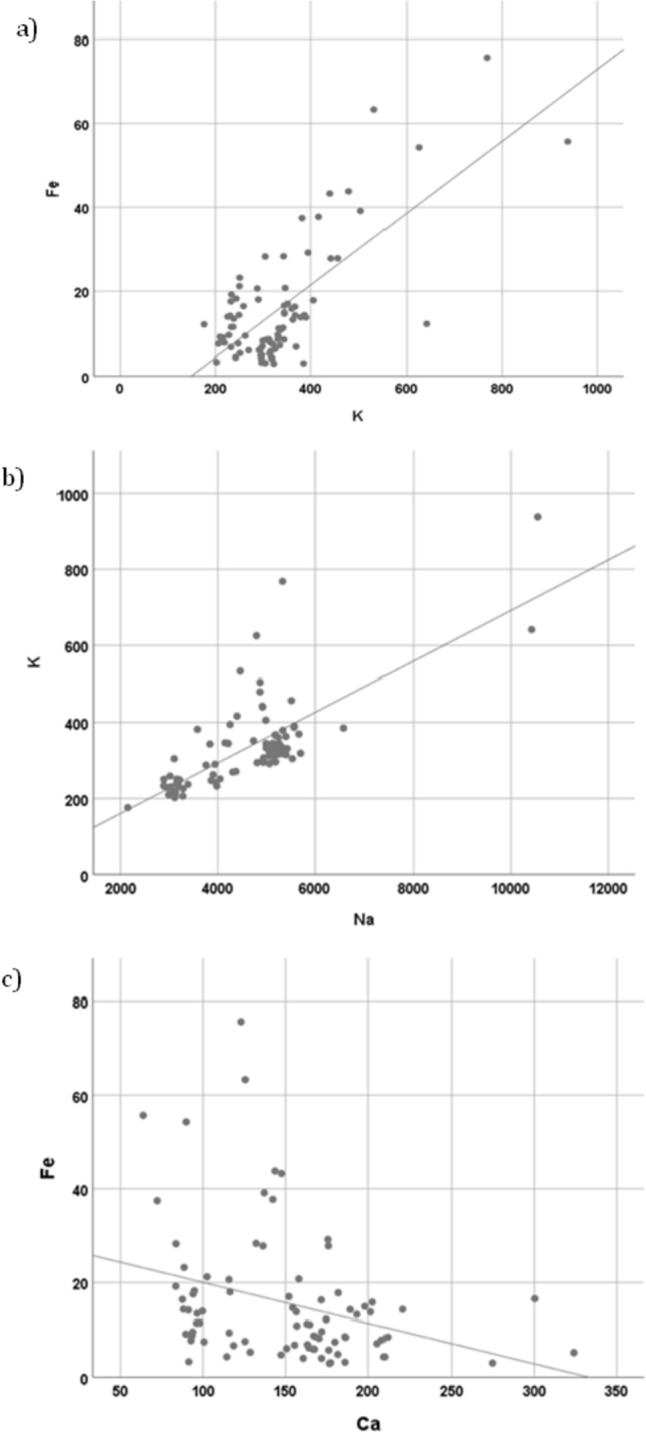
Correlation between **a**) K-Fe, **b**) Na-K, and **c**) Ca-Fe

Ca was positively correlated with Cu and Na, and negatively with Fe. This negative association indicates that when the Pb content increases, the Ca content decreases, this is since Pb interferes with Ca metabolism since they are chemically similar and Pb acts by displacing Ca. Ni did not show significant correlations with any metal (Fig. 2).

### FF and Metals in PCO

Twenty-two participants (24%) had PCO. The presence of metals in PCO patients was analysed. There were no significant differences in the characteristics of women or in the presence of metals according to PCO (Table 3).

**Table 3:** Characteristics and metal content according to PCO

	PCO		*p*-value
	Yes (*N* = 22)	No (*N* = 71)	
Age (years)	35.1 ± 4.5	36.3 ± 3.7	0.208
BMI (kg/m^2^)	25.1±3.3	24.5±3.4	0.457
Essential metals, *n* (%)			
Cu	19 (86)	66 (93)	0.388
Fe	20 (91)	68 (96)	0.589
Zn	19 (86)	66 (93)	0.388
Mn	12 (55)	46 (65)	0.453
Cu (mg/kg)	0.95 ± 0.45	0.86 ± 0.24	0.249
Cu ≤ 0.51	7 (32)	6 (9)	0.010
Fe (mg/kg)	17.87 ± 13.96	14.78 ± 13.90	0.385
Fe (mg/kg)*	14.2 (6.8; 26.0)	9.86 (7.0; 16.5)	0.282
Zn (mg/kg)	1.03 ± 0.37	1.17 ± 0.39	0.181
Zn ≤ 0.8	10 (46)	16 (23)	0.041
Mn (mg/kg)	0.04 ± 0.01	0.04 ± 0.01	0.319
Non-essential metals, *n* (%)			
Ba	2 (9)	9 (13)	0.938
Al	13 (59)	52 (73)	0.287
Al ≤ 0.2967	16 (73)	27 (38)	0.007
Ni	3 (14)	8 (11)	0.718
Pb	1 (5)	1 (1)	0.419
Sr	1 (5)	–	0.237
Ba (mg/kg)	0.03 ± 0,01	0.07 ± 0.07	0.488
Al (mg/kg)	0.81 ± 1.34	0.62 ± 0.35	0.358
Al (mg/kg)*	0.30 (0.26; 0.79)	0.54 (0.35; 0.80)	0.187
Ni (mg/kg)	0.17 ± 0.03	0.36 ± 0.48	0.517
Macroelements, *n* (%)			
Ca	18 (82)	67 (94)	0.087
Ca ≤ 176.62	21 (96)	52 (73)	0.035
K	19 (86)	67 (94)	0.350
K > 405.7167	6 (27)	5 (7)	0.019
Mg	3 (14)	7 (10)	0.696
Na	20 (91)	70 (99)	0.138
Ca (mg/kg)	139.5 ± 33.8	151.3 ± 52.7	0.373
K (mg/kg)	351.0 ± 131.1	327.6 ± 115.4	0.450
Mg (mg/kg)	70.9 ± 7.0	67.9 ± 7.0	0.548
Na (mg/kg)	4609 ± 1672	4540 ± 1185	0.836

^*^Median (interquartile range)

Using the cut-off point according to the Youden Index for the metals that are closest to significance (Table 3) suggests that low values of Cu, Zn, Al, and Ca favour PCO. The AUROC of this model is 0.673 (95% CI 0.546; 0.801; *p* = 0.014).

### Oocytes

Two of the ninety-three participants did not have mature oocytes, which means that the FR and CR were calculated based on a total of ninety-one interventions. The number of oocytes varied between one and thirty-three, with a median number per OPU of nine (5; 13).

An inverse correlation was also observed between the number of oocytes and the participant’s age (*r* = −0.353; *p* = 0.001). No correlation was observed between the number of mature oocytes and the FR.

The PCO participants presented a mean oocytes number of 14.3 (7.2) significantly higher (*p*<0.001) than the group of non-PCO women where the mean number of oocytes was 8.3 (5.1). However, no significant differences were observed between the number of oocytes and that of mature oocytes (*p*= 0.159), which was 2.1 in the PCO group (s.e. 0.25) and 1.4 (s.e. 0.30) in no PCO patients.

Regarding the relationship between oocyte number and metals: Fe (*r*_*s*_ =0.303; *p* =0.003) and Ca (*r*_s_ = −0.276; *p* = 0.007) were significant and Al (*r*_*s*_ =−0.195; *p* = 0.061) and Na (*r*_s_ = −0.174; *p* = 0.095) were close to significance.

The relationship between the number of mature oocytes and Fe (*r*_s_ =0.319; *p* = 0.002), Ca (*r*_s_ =-0.307; *p* = 0.003), and Na (*r*_s_ =−0.215; *p* = 0.039) were significant; and next to significance was Al (*r*_s_ =−0.198; *p* = 0.057).

### Fertilisation Rate

The median FR was 76.5% (52.6; 92.3). Twenty-one participants (23%) presented 100% FR and eight participants (8.8%) had an FR below 33.3%, with four participants (4.4%) having a FR of 0%. The median difference between mature oocytes and fertilised oocytes was 1 (0; 3), with this varying between 0 and 12.

**Fig. 3:**
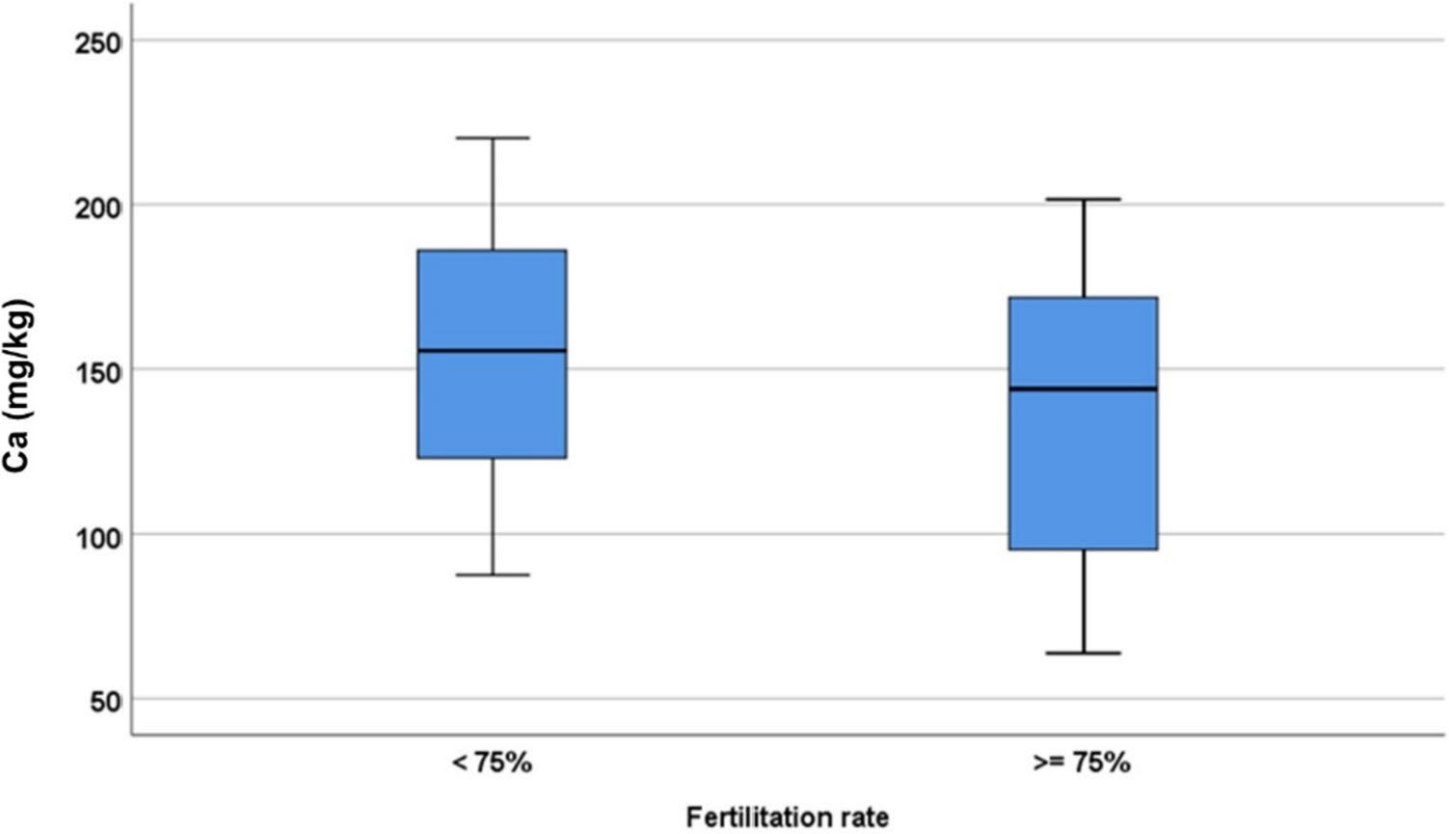
Fertilisation rate and Ca levels

High Ca impairs FR. Using the cut-off point according to the Youden index, ≤ 176.62 is obtained (*p*-value = 0.011). Ca above 176.62 mg/kg decreases FR (Fig. 3).

That is, if *p* = probability of having an FR ≥ 75% then ln *p*/(1−*p*) =−1.495+2.406*(*if* technique=ICSI) −1.630*(*if* Ca>176.6 mg/kg). The AUROC of this model was 0.687 (95% CI 0.564; 0.810; *p* = 0.004).

### Cleavage Rate

The CR was calculated in the group that had at least one fertilised oocyte. In this data set, there were four participants that did not achieve at least one fertilised oocyte and therefore the number of valid interventions for this calculation was eighty-seven. Seventy-seven interventions (88.5%) achieved 100% CR and only five participants (5.7%) had a rate ≤80%.

Regarding the availability of metals, no significant differences were found in any of them between the two CR groups. The classification of the spermiogram is also noteworthy, although not significant. In the group with <100% division, 70% presented pathology, compared to 39% in the group with 100% embryo division (*p* = 0.090).

For Cu, Fe and Al a study has been carried out, using the Youden index criterion, to determine a possible cut-off point, which could give significance. If Fe ≤ 7.525, Cu ≤ 1.04 and Al > 0.31 are presented with a value lower than that described, they are more likely to reach 100% CR. If *p* = probability of having a CR = 100%, then ln *p* / (1-*p*) =0.177+2.025*(if Cu≤1.04 mg/kg) +1.448*(if Al>0.31 mg/kg). The AUROC of this model was 0.799 (95% CI 0.648; 0.951; *p* = 0.002). An excess of Cu and Al are harmful.

### Embryo Quality

GEQs were obtained from the group that had at least one fertilised oocyte, that is, from eighty-seven participants. Their values varied between 0 and 9, with a median of 2 (1; 4). A GEQ was not achieved in eighteen (20.7%) participants, a single GEQ was achieved in sixteen (18.4%) participants, and five or more GEQs were achieved in seventeen (19.5%) participants. The rate exceeded 50% in half of the cases and reached a rate of 66.7% in 25% of the cases, although 25% had a GEQ rate of less than 20%. Seven participants reached a 100% GEQ rate (Table 4).

**Table 4:** Characteristics and metal content according to the GEQ rate

	GEQ		*p*-value
	< 66.6% (*N* = 63)	≥ 66.6% (*N* = 24)	
Female			
Age (years)	35.9 ± 3.6	36.4 ± 3.6	0.577
Weight (kg)	66.0 ± 10.3	64.4 ± 7.8	0.499
Height (m)	1.64 ± 0.08	1.63 ± 0.07	0.813
BMI (kg/m^2^)	24.6 ± 3.6	24.2 ± 3.0	0.599
BMI *n* (%)			0.356
Low/normal	34 (55)	14 (58)	
Overweight	23 (37)	10 (42)	
Obese	5 (8)	–	
PCO	16 (25)	6 (25)	1.000
Male			
Age (years)	37.8± 5.4	38.4±4.7	0.661
Weight (kg)	83.6 ± 13.2	86.2 ± 13.9	0.452
Height (m)	1.76 ± 0.05	1.76 ± 0.06	0.796
BMI (kg/m^2^)	27.0 ± 4.1	27.7 ± 4.3	0.508
Spermiogram			0.809
Normal	37 (59)	13 (54)	
Pathological	26 (41)	11 (46)	
Technique			0.997
IVF	4 (6)	1 (4)	
ICSI	59 (94)	23 (96)	
Metals			
Essential metals *n* (%)			
Cu	59 (94)	20 (83)	0.208
Fe	61 (97)	21 (88)	0.126
Zn	59 (94)	20 (83)	0.208
Mn	43 (68)	12 (50)	0.139
Cu (mg/kg)	0.87 ± 0.28	0.90 ± 0.33	0.685
Fe (mg/kg)	16.71 ± 14.42	13.10 ± 13.33	0.317
Fe (mg/kg)*	13.2 (7.5; 18.1)	9.4 (5.3; 15.4)	0.176
Fe > 10,043	37 (59%)	8 (33)	0.038
Zn (mg/kg)	1.15 ± 0.41	1.09 ± 0.36	0.595
Mn (mg/kg)	0.04 ± 0.01	0.04 ± 0.01	0.403
Non-essential metals, *n* (%)			
Ba	8 (13)	2 (8)	0.720
Al	46 (73)	15 (63)	0.433
Ni	9 (14)	2 (8)	0.720
Pb	1 (2)	–	0.724
Sr	–	1 (4)	0.276
Al (mg/kg)	0.68 ± 0.77	0.60 ± 0.32	0.676
Al (mg/kg)*	0.50 (0.30; 0.81)	0.49 (0.34; 0.89)	0.821
Ni (mg/kg)	0.36 ± 0.44	0.07 ± 0.02	0.406
Ni (mg/kg)*	0.16 (0.08; 0.54)	0.07 (0.06; 0.09)	0.098
Macroelements, *n* (%)			
Ca	59 (94)	20 (83)	0.208
Ca> 93.725	51 (81)	14 (58)	0.034
K	60 (95)	20 (83)	0.088
Mg	6 (10)	3 (13)	0.702
Na	62 (98)	22 (92)	0.183
Ca (mg/kg)	148.1 ± 47.2	140.0 ± 40.7	0.496
K (mg/kg)	337.9 ± 127.9	319.7 ± 103.7	0.567
Mg (mg/kg)	68.5 ± 7.5	70.9 ± 6.9	0.656
Na (mg/kg)	4534 ± 1201	4530 ± 1592	0.991

*Median (interquartile range)

If the Youden index criterion was used for the metals that are closest to being significant, a cut-off point > 10.043 would be obtained for Fe and a cut-off > 93.725 for Ca. If Fe>10.043, GEQ is less than 66.6%. Introducing these two variables in the logistic regression model (forward selection of Wald variables) would indicate that an excess of Fe (>10.043) and an excess of Ca (> 93.725) would reduce the GEQ rate. The AUROC of this model would be 0.627 (95% CI 0.569; 0.815) *p* = 0.006.

In the present study, high Ca and Fe levels were harmful to GEQ.

### Blastocyst Rate

Out of the total number of cycles, the culture was started in sixty-nine (79.3%) of the eighty-seven participants who presented embryos with FR of at least one cultured embryo by days 5–6. The median number of cultured embryos was three (1; 6). Twenty-seven participants (39.1%) presented 0% BR and nine participants (13%) presented 100% BR and a median of 33% (0; 50).

If the cut-off points are calculated following the Youden index, it is obtained that for Ca and K an excess of these metals impairs the BR. Applying logistic regression with these two variables, only K would remain in the model (of those with Ca > 170.335, 94 also have K > 304.7466 mg/kg).

The ROC curve shows an area of 0.641 (IC_95%_ 0.503; 0.779; *p* = 0.030).

Forty-eight participants had frozen embryos, in a total of sixty cycles (twelve of the participants had two frozen cycles). The median in this case was 75 (0; 100). Of these sixty cycles, blastocysts were not obtained in seventeen (28.3%) and 100% was achieved in twenty-eight (46.7%). Attempts were made to form groups with <50% and ≥50%, as well as equal to 100% or not, but in this case, it was not significant.

There was a total of 129 cycles (69 fresh and 60 frozen). The median BR was 50% (0; 100%). Forty-four cycles (34.1%) presented a BR of 0% while thirty-seven (28.7%) had a BR of 100%.

High Ca and K levels impaired BR and, on the contrary, low Ca levels improved BR. The presence of Mn was detrimental to BR, particularly in those with BR ≥ 50%, 91% had an Mn ≤ 0.035 mg/kg, compared to only 67% if BR < 50% (*p=*0.039).

### Embryo Transfer

In a total of 110 cycles, at least one embryo was transferred (52 of the 69 fresh and 58 of the 60 frozen). The total number of participants who performed at least one cycle was eighty, eighteen of whom underwent both fresh and frozen transfer (ECT), thirty-four only in fresh, and twenty-eight only in ECT. There were fifty-one participants (63.7%) who had only one cycle, twenty-eight (35%) two cycles, and only one (1.3%) three cycles.

In 62% of the cycles, a single embryo was transferred and in the remaining 38% two embryos were transferred. These proportions do not differ significantly between fresh and ECT (*p* = 0.653) (fresh two embryos were transferred in 40% of the cycles and in ECT in 36% of the cycles).

The IR did not differ significantly according to the number of embryos transferred (*p* = 0.915). In the case of ECT, implantation occurred in 32.4% of the cases, compared to 33.3% when two embryos were transferred. In the case of fresh embryos, the IR was 25.8% when one embryo was transferred and 23.8% in the case of two embryos being transferred (*p* = 0.870), while in the frozen embryos this was 37.8% when one embryo was transferred versus 42.9% when two embryos were transferred (*p* = 0.707).

### Implantation Rate

Among the 110 cycles with embryo transfer, no implantation occurred in seventy-four cycles (67.3%), one embryo was implanted in thirty-three cycles (30%), and two embryos were implanted in three cycles (2.7%). Although it is not significant, implantation was achieved in thirteen cycles (25%) in the case of fresh embryos compared to twenty-three cycles (39.7%) with frozen embryos (*p* = 0.110).

No differences were found regarding the technique between fresh and frozen embryos, ICSI was used in 94% of fresh and 91% of frozen embryos (*p* = 0.719).

Although it is not significant (*p* < 0.1), around 15% of those who did not implant, presented Ba and Mg, while in those implanted this percentage was only 3% (a single cycle).

If K>237.18 and Ca ≤ 147.32, this favours implantation. This model had an AUROC of 0.716 (95% CI 0.611, 0.822); *p* < 0.001. The overall accuracy of this model was 74.5% (83.8% of those not implanted and 55.6% of those implanted.

In the present study, high Fe and K and low Ca levels improved IR.

### Pregnancy Rate

Of the 110 cycles with ET, thirty-six resulted in pregnancy (two of which were multiple), thirteen of these (36.1%) were from fresh cycles and twenty-three (63.9%) were from ECT. In other words, of the fifty-two total fresh cycles with ET, thirteen (25.0%) resulted in pregnancy, compared to twenty-three (39.7%) out of the fifty-eight ECT cycles (*p* = 0.110). Of the thirty-six pregnancies, twenty-seven (70.0%) evolved positively (two twin pregnancies), eight (22.2%) ended in abortions, and one in an ectopic pregnancy. In the case of fresh cycles, ten (76.9%) of the thirteen pregnancies evolved positively (one twin) compared to ECT where seventeen (73.9%) evolved positively (one also with twins) (*p* = 0.981).

If the cut-off points (Youden index) of the variables that best discriminate between pregnancy success are used. This model has an AUROC of 0.741 (95% CI 0.539, 0.943).

PR is influenced by high Fe and K levels and low Cu levels.

## Discussion

### Follicular Fluid

FF is the fluid that surrounds the developing oocyte in the ovary and can be analysed to assess metal content as well as to determine potential exposure to toxic elements in women seeking ART [17]. Butts et al. [22], estimated that “compared to blood and urine specimens, FF provides a better estimate of exposures that might impact reproductive outcomes, as it more closely reflects the microenvironment surrounding the developing oocyte,” being a reasonable biomarker of the local microfollicular environment [23]. However, another clinical study [22] reported variation in metal levels in inter-follicles in the same woman, and some authors [24] found that element concentrations in small follicles frequently differed from those of large follicles. To get round this issue, FF from several follicles of the same patient, both small and large, was pooled and the mean average level of the total FF was measured in the present study.

### Metals

Essential metals (Cu, Fe, Zn) were present here in all the analysed FF samples, except for Mn (62%). On the other hand, non-essential metals (Ba, Ni, Pb, Sr) were less common, as expected since there was no environmental or occupational exposure, except for Al (70%) with a more frequent presence, Co, Mo, Cr, B, Cd, Li, and V were not detected and they could have been below the detection limit of the device (ICP-OES). Finally, the macroelements (Ca, K, Na), with the exception of Mg (11%), were present in most of the FF samples.

The proper comparison of the metal concentration with other authors was extremely difficult, not only because of the different detection methods used, but also, because of the different units used: mg/l [15, 23] or μg/L [17, 22, 25, 26].

Some authors observed that metal contents in FF were inconsistent, and most of them were lower in FF than in serum, except for Cr, Mn, Ni, and Pb [26]. In addition, low presence of metals except for the macroelement Ca, K, Mg, and Na, and Fe was found in the present study. These data could suggest a possible protective role against certain toxins of the follicle wall. However, Wu et al. [25] reported that most of the elements such as Cr, Ni, Cd, and Pb were highly detected (71.8–100%), except for Cr in FF, which had a detection rate of 66.0%.

Regarding these above mentioned metals, Cr and Cd were not detected in the present study, and Ni (12%) and Pb (2%) were very low, and no significant amounts of Pb in the FF of the patients studied were found, and this may be directly related to the fact that the environment in which the patients live is an island with a low level of industrialisation and pollution and, furthermore, the female participants in this study did not report environmental or occupational exposure.

### Association Between Metals and ART

Although there are few publications about the action of HM in FF and their influence on ART, several studies have shown that chronic metal exposure can affect not only the oocyte, but later the embryo development [11, 13]; while in the case of Cu, Cd, Pb, Zn, and Fe, some authors did not find any relationship [5] or reported a negative impact [3]. However, an influence of Ca, Cu, Fe, K, Mn, and Al on the ART results was observed in the present study.

Regarding the possibility that metal mixtures may have interactive effects (additive, synergistic or antagonistic) on human health, it is necessary to consider its potential effect on pregnancy outcomes, even at very low concentrations [2]. These metal mixtures may reflect a common source for the metals [27].

Niehoff et al. [28], reported the strongest correlation between Fe and Mn. Most authors studied the mixture of toxic elements such as Cd, Se, Hg, Pb, or As that were not detected in the present study.

### Association Between Cu and ART

Cu is a component of the antioxidant superoxide dismutase (SOD) metalloenzyme which neutralises superoxide radicals and prevents the formation of more reactive and harmful free radical species [29]. In addition, SOD activity is inversely associated with fertilisation [30].

But HM are not always associated with harmful effects, thus the average number of oocytes recovered was positively associated with higher urine Cu concentrations, embryos generated and more total embryos [13], also more fertilised oocytes [31], and more pregnancies [5] were found in patients with higher concentration of Cu.

On the other hand, several authors found no relationship between the FF Fe levels and the effect on A

RT and embryonic development or with PR [32].

Tolunay et al. [3] found no relationship between Cu concentration in FF and ART outcome. However, several authors, as is the case of the present study, found lower PR in patients with higher concentrations of Cu in FF, as well as a negative effect on CR, and found that for every 1 mg/dL higher concentration of Cu in the FF there is a 71.9% lower chance of pregnancy, with a possible negative effect on follicular maturation and embryonic development. Furthermore, Wodiak et al. [5] observed that the highest concentrations of Cu were accompanied by shorter times for each of the various embryo development stages, and as we know [20], alterations in embryonic morphokinetic harm reproductive results. The results of the present study show that Cu levels should be less than 1.04 mg/kg so as not to affect the CR. This negative effect on follicular maturation and embryo development is suggested when exposure to Cu is chronic [3].

### Association Between Fe and ART

Regarding the Fe outcome, this was positively correlated with ROS levels. Fe can be delivered to granulosa cells by soluble transferrin transporter, which captures Fe released in interstitial spaces [33].

It should be mentioned that in the results reported here, there was an inverse relationship between Ca and Fe, and lower concentrations of Ca were linked to a higher concentration of Fe and improved oocytes number and mature oocytes obtained.

It is known that when mature oocytes could be extracted, they would increase GEQ [34]. Although Fe may affect the development of the oocytes, it does not seem to affect the embryo quality, since no correlation was observed between ferritin levels in the follicles and embryo quality [5].

In the present study, Fe was related to GEQ. Thus, participants with Fe levels lower than 7.52 mg/kg in the FF had a higher CR and GEQ, and participants whose Fe levels in FF were higher than 14.32 mg/kg, presented worse values in the CR and poorer GEQ, despite having a better IR. In addition, when Fe concentration was higher than 0.0433 mg/kg, associated with a Ca concentration higher than 93.725 mg/kg, there was a detrimental effect on GEQ.

In summary, high levels of Fe were associated with low CR and low GEQ, although it improved the IR.

Several authors have not been able to demonstrate that ARTs were affected by the presence of Fe in blood or in FF [3], but in the present study we did.

### Association Between Ca and ART

In this study, low levels of Ca were observed to improve FR, IR and BR, while higher levels harmed FR, BR, GEQ rates. The concentration of Ca needs to be less than 93.72 mg/kg for the GQE to improve, and values below 170 mg/kg improves BR, while values not exceeding 147 mg/kg do not affect IR. FF Ca levels should not exceed 93.72 mg/kg to expect the best results in ART. On the other hand, Ca does not seem to be influencing CR.

In this regard, the important role of Ca^2+^ in oocyte maturation, fertilisation, and early embryo development is well known. For the homeostasis of Ca, the intracellular content is stored in the endoplasmic/sarcoplasmic reticulum (ER/SR), and extracellular Ca is also important for the proteins involved in Ca^2+^ signalling and transport. Human oocytes and embryos have a perfectly organised and diversified system for Ca sequestration and release [35]. Ca is essential to trigger oocyte activation. Several authors reported that FF Ca concentrations were higher in participants who did not achieve any pregnancy, the difference did not reach statistical significance level, compared to those who did; following ICSI attempts [36], this result does not clarify the role of Ca in ART.

Other authors postulated that a higher intracellular ovum concentration of Ca^2+^ could be represented as a higher FF level of Ca^2+^ [36]. In the ICSI cycle, the dysfunction of oocyte activation caused by insufficient release of Ca^2+^ from the endoplasmic reticulum is one of the main reasons for repeated fertilisation failure. Calcium ionophore (A23187) is a highly selective calcium ionophore, which can form a stable complex with Ca^2+^ and pass through the cell membrane, increasing intracellular Ca^2+^ levels, and can activate oocytes [37]. Furthermore, treatment with Ca^2+^ ionophore improved embryo development and reproductive outcomes in cases with previous developmental problems [38].

The present study shows that an elevated concentration of Ca in FF above 176.6 mg/kg, significantly affects the FR, pointing somehow to an imbalance in the homeostasis of Ca when extracellular Ca levels are exceeded in the FF during oocyte maturation. Another finding of this study is the confirmation that significantly smaller concentrations of Ca were recorded in the FF of PCO patients.

### Association Between K and ART

As regards K ions, we know they are involved in many important functions such as transmembrane transport, activation of glycolytic enzymes and synthesis of macromolecules [39], and a relationship is suggested with the Na+/K+ ATPase being directly implicated in blastocyst formation [40].

To the best of the authors’ knowledge here are no current studies on the subject, but it has been observed in animal research that increasing K concentrations in embryo culture media resulted in an increase in BR and morulae and blastocysts also had an increased number of cells indicating an acceleration in cell proliferation. This effect was reversed when the K concentration notably increased [39], a fact that is confirmed in the present study. A positive correlation between the concentration of K above 237.18 mg/kg, and the improvement of IR and PR was found. It should be mentioned that the K level was measured in the FF, with the K in culture media being directly related with embryo development and physiologic K measured in FF being more implicated in implantation processes.

Another noteworthy finding in the present study was that higher quantities of K above 405.71 mg/kg were associated with the PCO pathology.

### Association Between Al and ART

As for Al, which is a non-essential metal, humans are not immune from the burgeoning presence of Al in the biotic cycle. It is an unsafe metal whose presence is not banned in many countries and no legislation exists limiting Al human exposure. Nevertheless, humans are exposed to Al not only through food, but topically applied with cosmetics and hygiene products, especially anti-transpirants and sunscreens. Al is found in raw materials for the application of cosmetics due to their healing activity, hydration, pigment dispersion, and melanin adsorption. Al is also present in clay, but especially in the white clay used in cosmetics and it should be mentioned that Al may exert toxic effects when it reaches the circulatory system and accumulates in different organs [41], this would explain its presence in more than 70% of the women studied here did not report any environmental exposure. It is also used as medicine and vaccines adjuvant as well as in prostheses in surgery and dentistry [42], and it is important to be aware that human exposure to high concentrations of Al will result in toxicity although it is not common to find cases of intoxication from Al in the population. As the authors found in a previous study [19], elevated concentrations of Al in semen samples reduce GEQs that are related to the BR of the embryo cohort in ART. Along the same lines, when studying FF, a harmful influence of high Al presence was found on the CR in the embryo cohort, suggesting a negative effect of high amounts of Al on reproductive results. A relationship was found between Al and a lower number of obtained oocytes, but with a non-significant tendency. Al in FF should be below 0.31 mg/kg in order not to negatively influence the CR. This indicates the importance of having a convenient balance between exposure and excretion.

### Association Between Mn and ART

On the other hand, the participants with higher levels of Mn in urine had more oocytes retrieved and more embryos. Additionally, there was a relationship between obese and smoking women and lower trace elements implicated in anti-OS activities, such as Mn [43]. The present study found that Mn is detrimental to the BR, no influence on oocyte maturity was detected. Furthermore, the presence of Mn in the FF was negatively associated with the proportion of mature oocytes, and a lower likelihood for an oocyte to be recovered in metaphase-II arrest was detected. FF Mn was negatively associated with the rate of recovered mature oocytes [13].

### Association Between PCO and Metals

Several authors have demonstrated the relationship between metals and PCO [44]. The findings in the present study show that Al is present in more than 50% of PCO participants, even at low doses, and this is due to the impact of HMs on OS, which is a paramount cause in the aetiology of PCO, and OS and HM toxicity should be monitored in females to reduce the risk of PCO [44]. The PCO patient group was found to have lower levels of Cu, Zn, Al, and Ca but greater levels of K in FF.

It should be noted that the highest levels of Ca correlated with a decrease in the number of oocytes and in PCO patients the Ca level is lower when accompanied by the greatest number of oocytes which is a usual guideline in this pathology. The findings here show that participants with higher levels of Ca have lower oocyte numbers.

## Conclusions

Several metals detected in FF, such as Ca, Fe, Cu, Al and K, have an important influence on reproduction, and show that the analysis of metals opens up a new line of study on female infertility with implications in reproductive outcomes. Many cases of unknown infertility could be due to metals in FF [45], and treatments could be aimed at correcting possible anomalies in metals levels in infertile women.

Based on all the evidence, HM levels impact ART outcome, and controlling exposure to toxic elements for all couples with reduced fertility or receiving an ART is recommended. More studies with larger cohorts and different patient populations are needed to define their effects on oocyte quality and, therefore*,* on embryo development and human reproduction.

### Strengths and Limitations of the Study

It is known that environmental toxicants exposure affects human health. This prospective study analysed the association between twenty-two HM in infertile couples, and their effect on ART outcomes.

We conducted a long-term clinical screening; ninety-three couples were included in the present study. We tested the FF samples including twenty-two HM, combined with clinical data, and used statistical models to analyse the effects of the HM on the participants, the oocyte number and maturity, quality of embryonic development and pregnancy rates, among others. Our research will provide new tools for the prevention, diagnosis, and treatment of infertility.

Our subjects were all from different places. The geographical area where the participants come from are islands, and at the same time they come from different places on those islands, this gives us an idea of the factor geographic location and geographic differences and the test results may reflect the situation in a large area, although they have all been attended in the same reference hospital.

A strength of this work is that the FF of several follicles, both large and small, was pooled, considering that several authors [22] have detected variability in metal levels in inter-follicles in the same woman, and other authors [24] found that element concentrations in small follicles frequently differed from those in large follicles. To get round this issue, FF from several follicles was pooled and the mean average level of the total FF was measured in the present study.

We also analysed the impact of elements on the outcomes of ART and did consider the interference of factors such as living habits (smoking, drinking), or occupational exposure. Furthermore, our results are not vulnerable to confounding by factors related to exposure and outcome, such as related environmental exposures, health behaviours, or dietary factors that may be important and have been assessed. The limited number of previous studies characterising predictors of FF metals in infertility patients, and in humans in general, may limit our ability to assess impact.

Our data are only for these couples who need ART, and does not involve the general population, but it would be very difficult to perform follicular punctures on patients who are not going to undergo ART.

The study has some limitations and thus the results should be interpreted with caution. The participants did not refer to exposure to environmental or professional factors, and as such toxic metals as important as Pb and Hg have not been detected, and Al with a very low incidence and, therefore, we were unable to assess their importance. This prevented us from an exact assessment of the influence of HM toxic on ART.

We were also limited by the inability to stratify for infertility factors that are likely to modify the association between FF metal contents and ART outcomes, including diagnosis.

Other important limitations relate to the possibility of FF contamination by blood during the ovum pick up aspiration procedure. However, we excluded the red-coloured specimen, so we avoided that possible effect. In addition, we used a surgical grade stainless steel needle that could imply contamination of the samples but the non-detection of metals such as Pb, Hg, or Cd ruled out this possibility.

On the other hand, if we were to increase the sample size, type II errors would be reduced which is why this study mainly emphasises significant results. When the study was initially proposed, it was thought that the contents of the different metals evaluated would be compared in two independent groups defined on ART outcome. It was considered that the groups would be formed with a 1:4 design (because they were mainly defined by quantiles) and that the standardised difference in metal content to detect between groups would be 0.75 units with a confidence level of 95% and a power of 80%, including 5% for possible losses.

In fact, we consider this study as a preliminary basis to extend it, in the near future, to a more in-depth study of various pathologies within the different aetiologies of infertility.

Finally, it has not been possible to explain whether elements will specifically harm fertility, larger studies in the ART population are required to find the association between the outcomes of the treatment and HM, for future diagnostic and treatment protocols.

### Supplementary information

ESM 1: (DOCX 531 kb)

ESM 2: (DOCX 531 kb)

ESM 3: (DOCX 14 kb)

ESM 4: (PDF 98 kb)

ESM 5: (DOCX 18 kb)

## Data Availability

The data that support the findings of this study are available from the corresponding author upon reasonable request.
