# Supplementary figures and images for: The Influence of Follicular Fluid Metals on Assisted Reproduction Outcome
Source: Biol Trace Elem Res. 2023 Feb 18;201(11):5069–82. doi: 10.1007/s12011-023-03578-3 (PMC10509058; doi:10.1007/s12011-023-03578-3)

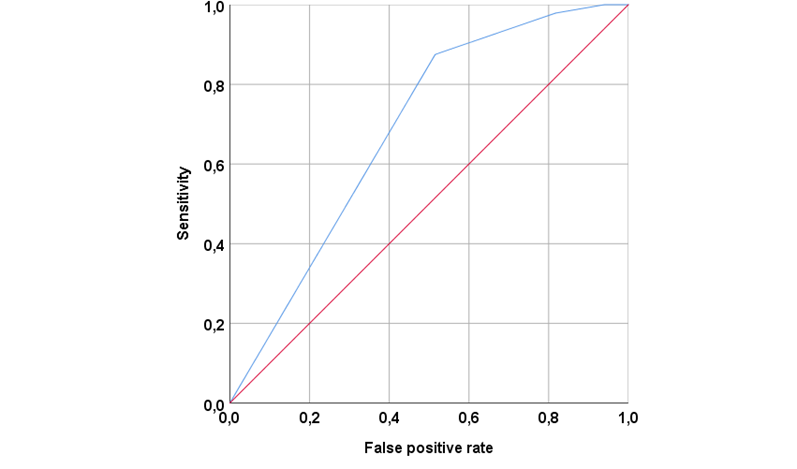


**0 0.2 0.4 0.6 0.8 1**

**0 0.2 0.4 0.6 0.8 1**

ROC curve for FR ≥ 75%


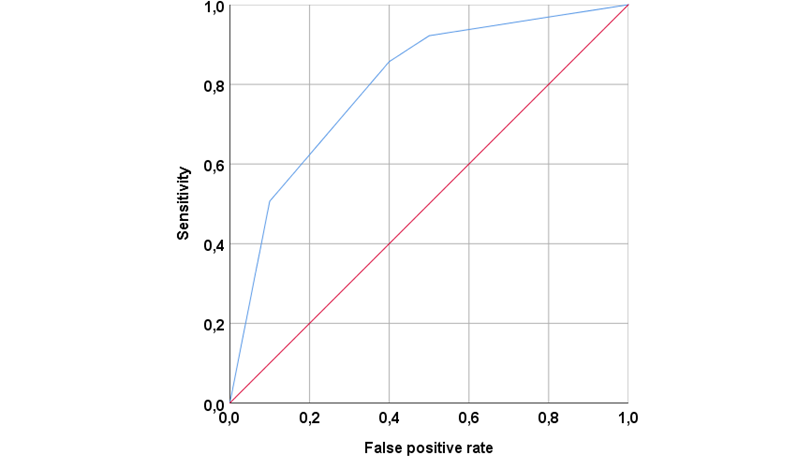


**0 0.2 0.4 0.6 0.8 1**

**0 0.2 0.4 0.6 0.8 1**

ROC curve for CR = 100%


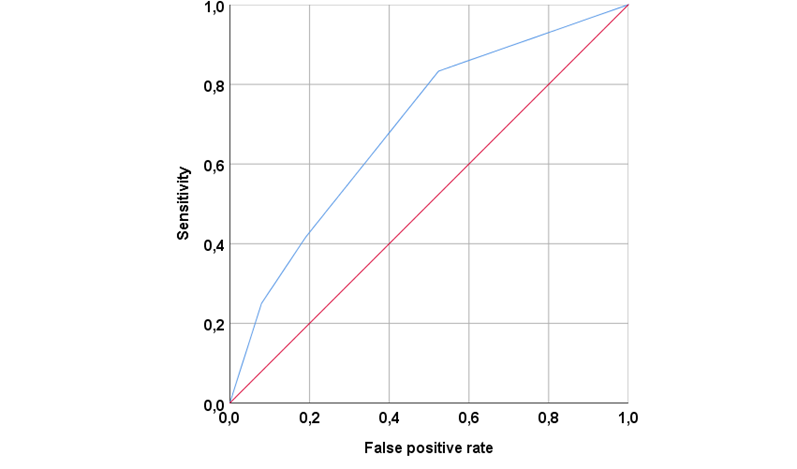


**0 0.2 0.4 0.6 0.8 1**

**0 0.2 0.4 0.6 0.8 1**

ROC curve of GEQ


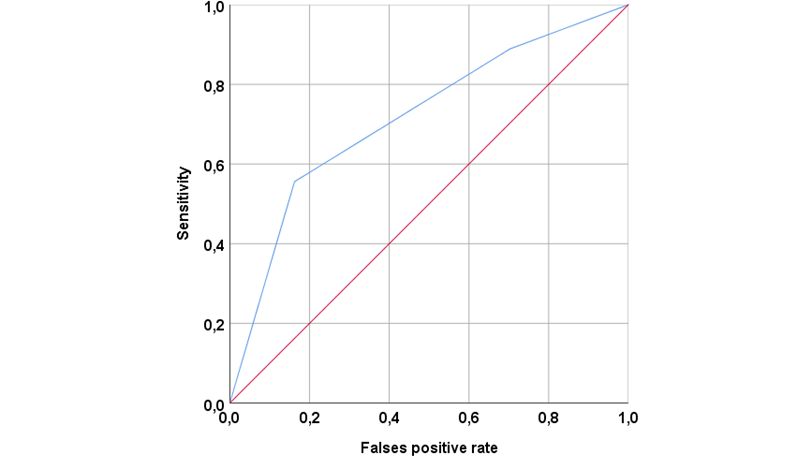


**0 0.2 0.4 0.6 0.8 1**

**0 0.2 0.4 0.6 0.8 1**

ROC curve of IR


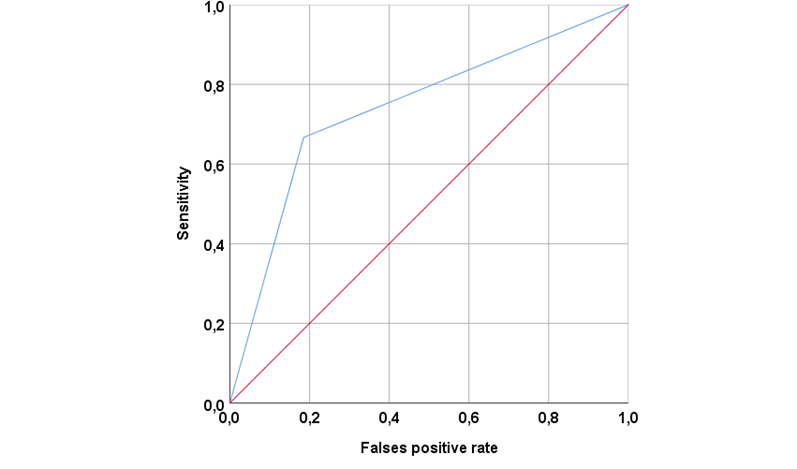


**0 0.2 0.4 0.6 0.8 1**

**0 0.2 0.4 0.6 0.8 1**

ROC curve for PR

Supplement: Supplementary file 1 — (DOCX 531 kb) [file 12011_2023_3578_MOESM1_ESM.docx]
