# Supplementary material for: The Influence of Follicular Fluid Metals on Assisted Reproduction Outcome
Source: Biol Trace Elem Res. 2023 Feb 18;201(11):5069–82. doi: 10.1007/s12011-023-03578-3 (PMC10509058; doi:10.1007/s12011-023-03578-3)
Supplement: Supplementary file 3 — (DOCX 14 kb) [file 12011_2023_3578_MOESM3_ESM.docx]

Digestion process temperature ramp information

| **No** | **Ramp** | **Temp** | **Keep** |
| --- | --- | --- | --- |
|  | **(mm:ss)** | **(°C)** | **(mm:ss)** |
| 1 | 15:00 | 50 | 5:00 |
| 2 | 5:00 | 60 | 4:00 |
| 3 | 5:00 | 70 | 3:00 |
| 4 | 3:00 | 90 | 2:00 |
| 5 | 20:00 | 180 | 10:00 |

instrumental parameters of the equipment

| **Instrumental Parameters** | |
| --- | --- |
| RF Power | 1150 W |
| Nebulizer Gas Flow | 0.50 L/min |
| Nebulizer Gas Pressure | 0.2 L/min |
| Auxiliary Gas Flow | 0.50 L/min |
| Cool Gas Flow | 12.5 L/min |
| Pump Speed | 45 rpm |
