# Supplementary material for: The Influence of Follicular Fluid Metals on Assisted Reproduction Outcome
Source: Biol Trace Elem Res. 2023 Feb 18;201(11):5069–82. doi: 10.1007/s12011-023-03578-3 (PMC10509058; doi:10.1007/s12011-023-03578-3)
Supplement: Supplementary file 4 — (PDF 98 kb) [file 12011_2023_3578_MOESM4_ESM.pdf]

HOJA DE INFORMACIÓN AL PACIENTE (1ª versión y 19 de julio de 2018)

TÍTULO DEL ESTUDIO: "ESTUDIO DE LA CALIDAD OVOCITARIA, EMBRIONARIA Y TASA DE FERTILIZACIÓN Y SU RELACIÓN CON LA PRESENCIA DE METALES EN LÍQUIDO FOLICULAR"

INVESTIGADOR PRINCIPAL Dra. Rubí Nieves Rodríguez Díaz, Unidad de Reproducción, Edificio Actividades Ambulatorias Hospital Universitario de Canarias, Ginecología 2ª planta, consulta número 11. (Teléfono 922678735).
CENTRO: HOSPITAL UNIVERSITARIO DE CANARIAS

INTRODUCCION

Nos dirigimos a usted para informarle sobre un estudio de investigación en el que se le invita a participar. El estudio ha sido aprobado por el Comité de Ética de la Investigación correspondiente.

Nuestra intención es tan solo que usted reciba la información correcta y suficiente para que pueda evaluar y juzgar si quiere o no participar en este estudio. Para ello lea esta hoja informativa con atención y nosotros le aclararemos las dudas que le puedan surgir después de la explicación. Además, puede consultar con las personas que considere oportuno.

PARTICIPACIÓN VOLUNTARIA

Debe saber que su participación en este estudio es voluntaria y que puede decidir no participar o cambiar su decisión y retirar el consentimiento en cualquier momento, sin que por ello se altere la relación con su médico ni se produzca perjuicio alguno en su tratamiento.

DESCRIPCIÓN GENERAL DEL ESTUDIO*:* Que conoce y acepta participar en el Estudio (código del protocolo):

"ESTUDIO DE LA CALIDAD OVOCITARIA, EMBRIONARIA Y TASA DE FERTILIZACIÓN Y SU RELACIÓN CON LA PRESENCIA DE METALES EN LÍQUIDO FOLICULAR"

Se realizará la determinación de metales en una muestra de líquido folicular, y se comparará con los resultados de la Fertilización in vitro (FIV). La finalidad es relacionar los resultados de la FIV con la presencia de metales, para valorar la influencia en los mismos. Los inconvenientes vienen derivados del tratamiento de reproducción y de la punción de FIV a la que usted se va a someter como parte de su tratamiento reproductivo, no suponiendo ningún riesgo añadido para la paciente. El líquido folicular que se obtiene en la punción de FIV y que normalmente se deshecha, en este caso será sometido a un análisis de metales. La paciente no va a obtener beneficio directo por su participación en el estudio, sino que contribuirá a ampliar los conocimientos en este campo de investigación.
Se realizará un total de 150 pacientes con esterilidad y comenzará en septiembre de 2018 y terminará en marzo de 2019.

El investigador principal será la Dra.: Rubí Nieves Rodríguez Díaz,

> Que se compromete a que cada sujeto sea tratado y controlado siguiendo lo establecido en el protocolo autorizado por el Comité Ético de Investigación Clínica.

Que respetará las normas éticas aplicables a este tipo de estudios.

Que dicho estudio se llevará a cabo contando con la colaboración de: Dr. Arturo Hardisson de la Torre y Dra. Raquel Blanes Zamora, como investigadores colaboradores.

CONFIDENCIALIDAD

Con la aplicación de la nueva legislación en la Unión Europea (UE) sobre datos personales, en concreto el Reglamento (UE) 2016/679 del Parlamento europeo y del Consejo de 27 de abril de 2016 de Protección de Datos (RGPD), es importante que conozca la siguiente información:

- Además de los derechos que ya conoce (acceso, modificación, oposición y cancelación de datos) ahora también puede limitar el tratamiento de datos que sean incorrectos, solicitar una copia o que se trasladen a un tercero (portabilidad) los datos que usted ha facilitado para el estudio. Para ejercitar sus derechos, diríjase al investigador principal del estudio. Le recordamos que los datos no se pueden eliminar, aunque deje de participar en el estudio para garantizar la validez de la investigación y cumplir con los deberes legales y los requisitos de autorización de medicamentos. Así mismo tiene derecho a dirigirse a la Agencia de Protección de Datos si no quedara satisfecho.

- Tanto el Centro como el Promotor y el Investigador son responsables respectivamente del tratamiento de sus datos y se comprometen a cumplir con la normativa de protección de datos en vigor. Los datos recogidos para el estudio estarán identificados mediante un código, de manera que no se incluya información que pueda identificarle, y sólo su médico del estudio/colaboradores podrá relacionar dichos datos con usted y con su historia clínica. Por lo tanto, su identidad no será revelada a ninguna otra persona salvo a las autoridades sanitarias, cuando así lo requieran o en casos de urgencia médica. Los Comités de Ética de la Investigación, los representantes de la Autoridad Sanitaria en materia de inspección y el personal autorizado por el Promotor, únicamente podrán acceder para comprobar los datos personales, los procedimientos del estudio clínico y el cumplimiento de las normas de buena práctica clínica (siempre manteniendo la confidencialidad de la información).

El Investigador y el Promotor están obligados a conservar los datos recogidos para el estudio al menos hasta 25 años tras su finalización. Posteriormente, su información personal solo se conservará por el centro para el cuidado de su salud y por el promotor para otros fines de investigación científica si usted hubiera otorgado su consentimiento para ello, y si así lo permite la ley y requisitos éticos aplicables.

Si realizáramos transferencia de sus datos codificados fuera de la UE a las entidades de nuestro grupo, a prestadores de servicios o a investigadores científicos que colaboren con nosotros, los datos del participante quedarán protegidos con salvaguardas tales como contratos u otros mecanismos por las autoridades de protección de datos. Si el participante quiere saber más al respecto, puede contactar al/ a la Delegado de Protección de Datos del Promotor.

INFORMACIÓN ADICIONAL

Tal y como exige la ley, para participar deberá firmar y fechar el documento de consentimiento informado.

El investigador principal de este estudio en este centro es la Dra. Rubí Nieves Rodríguez Díaz
Si durante la realización de este estudio le surge alguna cuestión relacionada con él puede consultar con la Dra. Rubí Nieves Rodríguez Díaz de la Unidad de Reproducción del hospital Universitario de Canarias

** Nota aclaratoria: en este documento aparecen en letra normal los aspectos que quedan fijos para todos los estudios, y en cursiva los aspectos variables dependiendo de las características del estudio, pero que deben obligatoriamente cumplimentarse.

CONSENTIMIENTO INFORMADO

Yo (nombre y apellidos)

....

He leído la hoja de información que se me ha entregado.
He podido hacer preguntas sobre el estudio.
He recibido suficiente información sobre el estudio.

He hablado con:

La Dra. Rubí Nieves Rodríguez Díaz.

Comprendo que mi participación es voluntaria.
Comprendo que puedo retirarme del estudio:

1º Cuando quiera
2º Sin tener que dar explicaciones.
3º Sin que esto repercuta en mis cuidados médicos.

- Presto libremente mi conformidad para participar en el estudio y doy mi consentimiento para el acceso y utilización de mis datos en las condiciones detalladas en la hoja de información.

Firma del paciente:
Nombre:
Fecha:

Firma del investigador:
Nombre: Dra. Rubí Rodríguez Díaz
Fecha:
