# Supplementary material for: The Influence of Follicular Fluid Metals on Assisted Reproduction Outcome
Source: Biol Trace Elem Res. 2023 Feb 18;201(11):5069–82. doi: 10.1007/s12011-023-03578-3 (PMC10509058; doi:10.1007/s12011-023-03578-3)
Supplement: Supplementary file 5 — (DOCX 18 kb) [file 12011_2023_3578_MOESM5_ESM.docx]

Logistic regression model for the probability of having PCO

| **Equation variables** | | | | | | | |
| --- | --- | --- | --- | --- | --- | --- | --- |
|  | Coefficient | s.e. | Wald | *p* value | OR | 95% C.I. OR | |
|  |  |  |  |  |  | Lower | Higher |
| Al ≤ 0.2967 | 1.469 | 0.538 | 7.471 | 0.006 | 4.346 | 1.515 | 12.462 |
| Constant | -1,992 | 0.435 | 20.960 | <0.001 | 0.136 |  |  |

Logistic regression model for the probability of having PCO

| **Equation variables** | | | | | | | |
| --- | --- | --- | --- | --- | --- | --- | --- |
|  | Coefficient | s.e. | Wald | *p* value | OR | 95% C.I. OR | |
|  |  |  |  |  |  | lower | higher |
| K ≤304.74666 | 1.178 | 0.543 | 4.704 | 0.030 | 3.248 | 1.120 | 9.418 |
| Constant | -1.350 | 0.424 | 10.130 | 0.001 | 0.259 |  |  |

Logistic regression model for the probability of being successful in the implantation

| **Equation variables** | | | | | | | | |
| --- | --- | --- | --- | --- | --- | --- | --- | --- |
|  | | Coefficient | s.e. | Wald | *p* value | OR | 95% C.I. OR | |
|  |  |  |  |  |  |  | Lower | Higher |
|  | K>237.18(1) | 2.216 | .655 | 11.448 | 0.001 | 9.167 | 2.540 | 33.082 |
|  | Ca≤147.32(1) | 1.715 | .492 | 12.168 | <0.001 | 5.556 | 2.120 | 14.560 |
|  | Constant | -3.420 | .733 | 21.770 | <0.001 | 0.033 |  |  |

Logistic regression model for the probability of having a successful pregnancy

| **Equation variables** | | | | | | | |
| --- | --- | --- | --- | --- | --- | --- | --- |
|  | Coefficient | s.e. | Wald | *p* value | OR | 95% C.I. OR | |
|  |  |  |  |  |  | lower | higher |
| Cu≤0.99 | 2.175 | 0.863 | 6.345 | 0.012 | 8.800 | 1.620 | 47.798 |
| Constant | -1.992 | 0.615 | 10.480 | 0.001 | 0.136 |  |  |
